# Acute non-traumatic abdominal pain presenting to emergency unit of a university teaching hospital in Rwanda

**DOI:** 10.1016/j.afjem.2025.100895

**Published:** 2025-08-08

**Authors:** Faustin Turamyimana, Jean Paul Dushime, Appolinaire Manirafasha, Deninson Martin Kyle, Doris Lorette Uwamahoro, Francois Regis Twagirumukiza, Pascal Mugemangango, Seraphina Negash, Anna Dobbins

**Affiliations:** a Department of Emergency Medicine and Critical Care, University of Rwanda, Kigali, Rwanda; b Department of Accident and Emergency Medicine, King Faisal Hospital, Kigali, Rwanda; c Accident and Emergency Department, University Teaching Hospital of Kigali, Rwanda; d Brown University, RI, USA

**Keywords:** Abdominal pain, Non-traumatic abdominal pain, Acute abdomen, Outcome, Triage, Rwanda

## Abstract

**Background:**

Emergency Department consultation for non-traumatic abdominal pain is one of the common reasons for presentation; accounting for 5.76–20 % of all Emergency Department consultations. Research about non-traumatic abdominal pain is limited in Rwanda and East Africa. This study aims to understand the clinical profile and outcomes of non-traumatic abdominal pain at the largest tertiary hospital in Rwanda.

**Methodology:**

A prospective cohort study of patients presenting with non-traumatic abdominal pain was undertaken.

**Results:**

During the 5-month study, two hundred sixty-one patients were enrolled in the study. The mean age was 39.7 years and male patients accounted for 57.5 % of the cohort. Nearly half of the cohort were triaged as a high priority (11.1 % red, 31.8 % orange), and 42.9 % were hemodynamically unstable at or shortly after presentation. More than half of the patients (57 %) had surgical conditions, including 40.2 % who underwent surgery and 17.6 % who were treated conservatively. The most common diagnoses were intestinal obstruction (25.7 % of all cases) and hollow viscus perforation (18.8 %). Mortality was 11.1 %, and the mean hospital length of stay was 9.1 days. Predicting factors for death outcome (p-value<0.05) were advanced age, altered mental status, jaundice at presentation, peritonitis, known malignancy, and acute kidney injury

**Conclusion:**

Abdominal pain is a common presenting problem, accounting for approximately 1 in 10 patients presenting to a tertiary care centre in Rwanda, with 2 in 5 patients requiring operative interventions. Identification of potential risk factors for mortality requires a multidisciplinary approach to decrease mortality and morbidity.

## African Relevance

• Non-traumatic abdominal pain is a common and understudied emergency in Africa.
• The study highlights region-specific causes and outcomes in an African setting
• Mortality remains high, emphasizing the need for early diagnosis and intervention.
• Findings support the development of targeted protocol for emergency care in Africa.
• The study addresses gaps in non-traumatic abdominal pain research on the continent.

## Introduction

In Rwanda acute abdominal pain is a common presentation complaint and reason for presenting to an Emergency Department (ED), accounting for 8.5 % of all emergency consultations in one East African country [1]. Etiology and mortality associated with non-traumatic abdominal pain vary by region [[1], [2], [3]]. Underlying causes for the pain vary amongst East African countries, with causes such as malignancy, intestinal obstruction, and peptic ulcer disease being most prevalent [1]. In high income countries, non-specific abdominal pain and ureteric colic are the leading causes [2,[4], [5], [6]]. Mortality rates also differ, ranging from 3.3 % in Germany [7], 5 % in the USA [6], and 8 % in Tanzania [1].

Limited data exist on the clinical profile, etiologies, outcomes, and factors associated with mortality in Rwanda. This study aims to describe the cohort presenting to a tertiary care hospital in East Africa with non-traumatic abdominal pain and determine factors that influence the risk of in-hospital mortality.

## Materials and methods

### Research setting

The University Teaching Hospital of Kigali ('Centre Hospitalier Universitaire de Kigali' – CHUK) is the largest tertiary care hospital in Rwanda, with 519 inpatient beds. CHUK is a referral for district hospitals in Rwanda and receives patients requiring a higher level of care, including those referred from district hospitals, self-reported patients, and those brought by Emergency Medical Services. The Emergency (ED) Department receives all patients, referred from other hospitals, or self-referrals, and treats approximately 20,000 patients each year. The ED is staffed with a mix of house officers supervised by consultants in Emergency Medicine. Patients are assessed and stabilized, and if necessary, subsequently referred to specialty services. Nurses triage all arriving patients using a locally modified South Africa triage scale [8].

### Study procedure

Patients presenting to the ED department at CHUK between October 1, 2023, to February 29, 2024, were prospectively screened for eligibility and inclusion in the study. Inclusion criteria were those aged 16 years or older with a primary complaint of non-traumatic abdominal pain and who provided consent for the study. Those experiencing cardiac arrest, who were pregnant, or who refused to provide consent were excluded.

The data regarding age, sex, medical history, triage score, findings from physical examination, investigations results, disposition, complications, management, and outcomes were collected using a questionnaire. Only those patients with a final diagnosis of non-traumatic abdominal pain, confirmed by the attending staff physician, were included. Data were collected from patients, charts, electronic files, and investigation reports by the research team, which included emergency physicians, residents, general practitioners, medical students, and nurses. Patients who were admitted to the hospital were followed until hospital discharge, while those discharged directly from the ED department were not followed up once in the community. The primary outcome of interest was mortality, with hospital length of stay and factors influencing mortality as secondary outcomes.

### Data analysis

Data analysis was completed using the Jamovi platform (https://www.jamovi.org/). A descriptive analysis to summarize demographics, clinical profiles, etiologies, and outcomes was performed. A binomial logistic regression between clinical profile characteristics and diagnoses and outcome (death versus survival), with linear regression between clinical profile characteristics and diagnosis and prolonged hospital stay of interest, to determine predictors of prolonged hospital stay. Analysis was conducted at a 95 % confidence level, with p-values of <0.05 considered statistically significant.

Ethical approval was obtained from the Institutional Review Board of the College of Medicine and Health Sciences, University of Rwanda (Approval notice: No 370/CMHS IRB/2023).

## Results

**Fig. 1:**
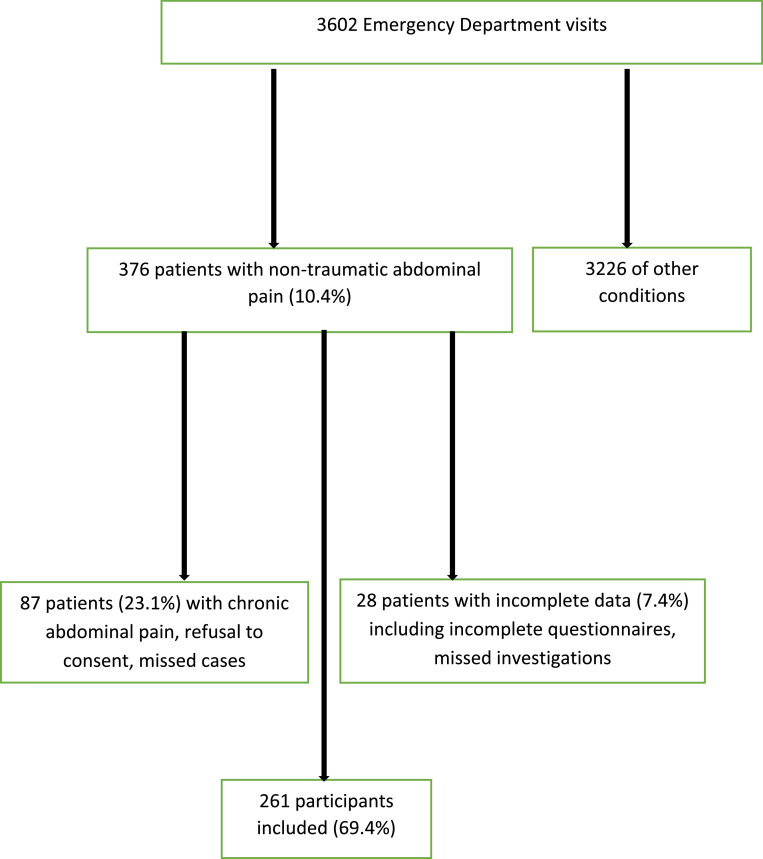
Identification of Cohort and Subject Recruitment.

During the study period, a total of 3602 patients presented to the Emergency Department(ED). Among these, 376 patients (10.4 %) reported abdominal pain. After applying inclusion and exclusion criteria, 261 patients (69.4 %) were included in the analysis ( Fig. 1).

Of these 261 patients, 57.5 % were male (150) and 42.5 % were female (111). The mean age of patients was 39.7 years (± 18.1 standard deviation) (Table 1).

**Table 1:** Patient stability, associated symptoms, past social, medical & surgical history, and examination findings.

Triage category	N	%
Orange category	83	31.1 %
Red Category	29	11.1 %
Unstable patient on arrival	112	42.9 %
**Associated symptoms**		
Abdominal distension	111	42.5 %
Non-bilious vomiting	111	42.5 %
Constipation	104	40 %
Gas arrest	98	38 %
Fever	57	22 %
Bilious vomiting	25	10 %
Weight loss	18	7 %
Headache	17	7 %
Jaundice	15	6 %
Dysuria	16	6 %
Cough	13	5 %
Lower Limbs swelling	10	4 %
Altered mental status	9	3.4 %
**Social History**		
Alcohol	44	16.9 %
Smoking	25	9.7 %
Herbal Medicine	6	2.3 %
**Past Surgical History**		
Abdominal surgery	29	11.1 %
**Past Medical History**		
Peptic ulcer disease	25	9.7 %
Hypertension	12	4.6 %
Diabetes Mellitus	7	2.7 %
Retroviral disease	7	2.7 %
Hepatitis	2	0.8 %
**Examination findings**		
Abdominal distension	127	48.7 %
Diffuse abdominal tenderness	118	45.2 %
Epigastric tenderness	60	23 %
Rebound tenderness	50	19.2 %
Right upper quadrant tenderness	36	13.8 %
Hypogastric tenderness	32	12.3 %
Right lower quadrant tenderness	29	11.2 %
Left lower quadrant tenderness	25	9.6 %
Left Upper Quadrant tenderness	11	4.2 %

The majority of patients (146; 55.9 %) presented with three or more associated symptoms (Table 2).

**Table 2:** Outlines the underlying causes of non-traumatic abdominal pain.

Etiology	Count(N)	Percentage
Intestinal obstruction	67	25.7 %
Generalized peritonitis	49	18.8 %
Non-specific abdominal pain	27	10.3 %
Peptic ulcer disease	21	8.0 %
Biliary and liver diseases	18	6.9 %
Malignancy	17	6.5 %
Gastro-enteritis	17	6.5 %
Simple appendicitis	9	3.4 %
Urinary tract infection	8	3.1 %
Pancreatitis	8	3.1 %
Adnexal masses	5	1.9 %
Intra-abdominal abscess	4	1.5 %
Other causes	11	4.2 %

The management and disposition of acute non-traumatic abdominal pain varied depending on the underlying cause and involved different clinical teams. The acute care surgery team managed most patients, often requiring abdominal surgery (laparotomy), particularly for conditions like intestinal obstruction, peritonitis, and appendicitis. This group had the highest number of deaths, especially among patients with intestinal obstruction and peritonitis (Table 3).

**Table 3:** Outlines patient management and outcomes (death versus survival).

Disposition	Type of management	Confirmed Diagnosis	N	Deaths, N (%)
Acute care surgery management	Laparotomy	Intestinal obstruction	38	10(3.8)
		peritonitis	41	
		malignancy	2	
		Appendicitis	2	
		Intra-abdominal abscess	1	
		Adnexal mass	1	
		pancreatitis	1	
		dolichocolon	1	
	Rigid sigmoidoscopy	Intestinal obstruction	3	0
	Herniorrhaphy	Hernia with Intestinal Obstruction	2	0
	Laparoscopy appendectomy	Simple appendicitis	4	0
	Conservatively managed	Intestinal obstruction	24	0
		peritonitis	7	6(2.2)
		cholecystitis	4	1(0.4)
		Appendicitis	3	0
		Intra-abdominal abscess	1	0
		pancreatitis	1	0
	Percutaneous abscess drainage	Intra-abdominal abscess	2	0
		Liver abscess	1	0
Urology	Simple nephrectomy	Renal abscess	3	0
	Ureteroscopy	Ureteric colic	1	0
	Bilateral ureteric stenting	Ureteric colic	1	0
Gynecology	Transferred to gynecology	Adnexal mass	4	0
		Tubo-ovarian abscesses	2	0
		Ruptured ectopic pregnancy	1	0
Internal medicine	Spontaneous bacterial peritonitis		1	1(0.4)
	Malignancy		15	8(3.06)
	Biliary/cholecystitis/liver abscess		13	1(0.4)
	Peptic ulcer disease		21	0
	Urinary tract infection		5	1(0.4)
	Ureteric colic		5	0
	Nonspecific abdominal pain		27	0
	Gastro-enteritis		17	1(0.4)
	Pancreatitis		6	0

### Complications at presentation

Among patients with non-traumatic abdominal pain, 35.2 % (92) presented, with signs of various organ dysfunctions upon arrival. The most predominant dysfunctions were acute kidney injury in 21.5 % (56), respiratory distress in 13.3 % (35), and acute liver injury in 9.6 % (25). Potassium abnormality and altered mental status were each observed in 3.4 % (9), while septic shock was the least common, occurring 1.1 % (3).

### Outcome measures

Of the 261 patients included in our study, 29 (11.1 %) died, with most deaths occurring within the first week of admission (20 out of 29). The mean length of hospital stay was 9.1 days (standard deviation ±11.5), with a maximum of 80 days.

When comparing the length of hospital stay and the timing of death, the data revealed that, a significant proportion of patients died within the first week of hospitalization. Of the 29 patients who had in-hospital deaths, 20 (7.7 % of the total sample) died within the first 7 days after admission.

The remaining nine patients died over the subsequent six weeks of hospitalization: one in the second week, one in the third, twos in the fourth, three in the fifth, and two in the sixth and seventh weeks.

**Fig. 2:**
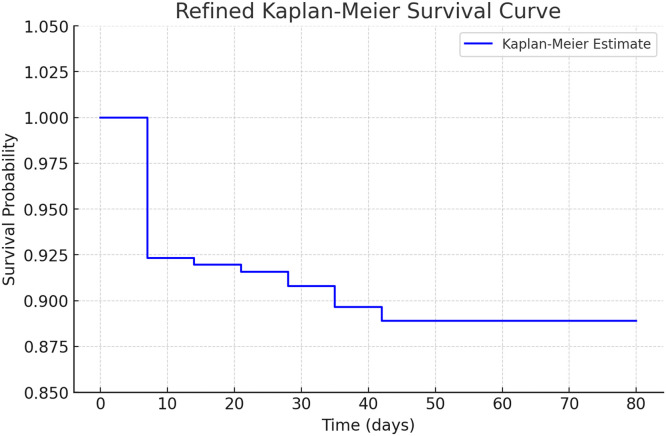
Kaplan-Meier survival curve for participants with acute non-traumatic abdominal pain.

The longest hospital stay was 80 days, while the longest stay among those who died was 44 days (Fig. 2). We found a steep drop in survival during the first week of hospitalization. Kaplan-Meier analysis of survival probability is provided as Supplementary Figure 2.

An Analysis of factors predicting poor outcome (death versus survival) was performed using binomial logistic regression. Independent predictors of in-hospital death are presented in Table 4.

**Table 4:** Predictors of in-hospital deaths.

Factors associated with mortality	Odd ratio	P-value	95 % confidence intervals
Age of 75 and above	6.18	0.021	1.31–29.2191
Malignancy	6.33	0.002	1.94–20.6804
Acute Kidney Injury	4.87	0.002	1.77–13.4482
Peritonitis	7.23	<0.001	2.46–213,026
Altered Mental Status	9.38	0.016	1.52–57.7274
Jaundice	13.33	<0.001	3.36–52.8265

Analysis of independent predictors of prolonged hospital stay showed that certain clinical signs and conditions were associated with longer hospitalization among patients with acute non-traumatic abdominal pain. These included older age (≥ 65 years), bloody diarrhea, and jaundice (Table 5).

**Table 5:** Predictors of prolonged hospital stay.

Factors Associated with prolonged hospital stay	p-value	95 % confidence intervals
65 years old and above	0.039	(−7.47) - (−0.1900)
Bloody diarrhea	<0.001	12.24 - 43.9346
Jaundice	0.044	(−12.30) – (−0.1802)
Hematochezia	<0.001	(−36.07) – (−10.5872)
Respiratory distress	0.048	(−6.57) – (−0284)
Left upper quadrant tenderness	0.012	(−14.46) – (−1.8020)
Acute Kidney Injury	<0.001	(−9.65) – (−2.9530)
Acute liver injury	0.019	(−10.76) – (−0.9601)

## Discussion

Acute abdominal pain was a common cause of visits to ED at Rwanda's largest hospital, accounting for 10.4 % of all visits as asserted in this study. Surgical conditions such as appendicitis and peritonitis are the most common causes of non-traumatic abdominal pain in sub-Saharan Africa and parts of Asia [[9], [10], [11]]. Our study found that surgical conditions, particularly intestinal obstruction and peritonitis, were the most common causes of non-traumatic abdominal pain. Similar findings were noted from Cameroon (10) where peritonitis and intestinal obstruction were most common. In contrast to a study from Nigeria (11), our findings showed that appendicitis was less common. Our findings also contrasted with high income countries and parts of Asia where non-surgical conditions such as nonspecific abdominal pain and ureteric colic were more common, and surgical acute abdominal pain was comparatively uncommon [3,4,6].

This study revealed an overall mortality rate was 11.1 %, with 7.7 % of deaths occurring in the first week of admission. This rate is comparable to another East African country (Tanzania) [1], but is much higher than reported in Germany [7] and the United States of America [6]. This study cannot determine the causes or contributing factors to this high overall rate. However, possible causes or explanations for this difference may include socioeconomic factors, availability of timely access to definitive care, health-seeking behavior, referral patterns, and availability of means of transport, amongst others.

### Limitations of the study

This study is limited due to possible selection bias, where the most severely ill are unable to provide consent. If included, these patients may ultimately increase the cohort's overall mortality. The study was only able to enroll just over two-thirds of potentially eligible patients. It is unclear if there are any differences between those enrolled and those who were eligible but not enrolled. This study is a single-centre study based at a tertiary care teaching hospital, and the study cohort is not likely to be a representative sample of the population as a whole. The cohort's comorbidities are not described, making it impossible to consider comorbidities as potential factors in or influences on survival. Finally, those discharged from the A&E department were not followed up after discharge. This lack of post-discharge information, including subsequent returns to the ED, complications, or deaths in the community, cannot be assessed.

## Conclusion

Abdominal pain accounts for 10.4 % of presentations to an ED in Rwanda, with just two in five requiring surgical intervention. One in nine patients died with advanced age above 75, acute kidney injury, jaundice, altered mental status, peritonitis, and malignancy being significant risk factors for death. To reduce these deaths, clinicians should implement strategies to identify high-risk patients early and ensure timely optimization, followed by urgent referral to appropriate specialty or early surgical intervention before clinical deterioration occurs. This approach should be supported by the development of standardized protocols for conditions such as acute kidney injury, acute non-traumatic abdominal pain, particularly peritonitis and cases involving elderly patients, who should be prioritized as a high-risk group. Additionally, the integration of palliative care should be enhanced. Strengthening human resources and infrastructure is also critical, including ensuring 24/7 availability of operating rooms, adequately trained staff in the ED, surgical teams, and sufficient critical care capacity.

## Dissemination of results

The results of this study will be disseminated through various channels to reach healthcare professionals, policymakers, researchers, and the public. Findings have been presented at the international conference, AFCEM2024. Locally, results will be shared with the Ministry of Health, CHUK leadership, and clinical teams to inform policy and practice. In addition, community will be engaged through public health campaigns and media summaries will highlight the importance of early treatment for abdominal pain. Additionally, we will collaborate with academic and healthcare institutions to integrate these findings into training programs and clinical protocols to improve patient outcomes.

## CRediT authorship contribution statement

**Faustin Turamyimana:** Conceptualization, Methodology, Project administration, Investigation, Data curation, Formal analysis, Resources, Writing – original draft, Writing – review & editing, Visualization, Funding acquisition. **Jean Paul Dushime:** Supervision, Methodology, Validation, Formal analysis, Visualization, Writing – original draft, Writing – review & editing. **Appolinaire Manirafasha:** Validation, Writing – original draft. **Deninson Martin Kyle:** Validation, Writing – original draft. **Doris Lorette Uwamahoro:** Validation, Writing – original draft. **Francois Regis Twagirumukiza:** Validation, Investigation. **Pascal Mugemangango:** Validation, Investigation. **Seraphina Negash:** Methodology, Formal analysis, Validation, Writing – original draft. **Anna Dobbins:** Software, Methodology, Resources.

## Declaration of competing interest

The authors declare that they have no known competing financial interests or personal relationships that could have appeared to influence the work reported in this paper.

## Appendix Supplementary materials

Image, application 1

Image, application 2

## Footnotes

Supplementary material associated with this article can be found, in the online version, at doi:10.1016/j.afjem.2025.100895.
