# Supplementary material for: Acute non-traumatic abdominal pain presenting to emergency unit of a university teaching hospital in Rwanda
Source: Afr J Emerg Med. 2025 Aug 8;15(3):100895. doi: 10.1016/j.afjem.2025.100895 (PMC12356451; doi:10.1016/j.afjem.2025.100895)
Supplement: Supplementary file 2 [file mmc2.pdf]

Supplemental material

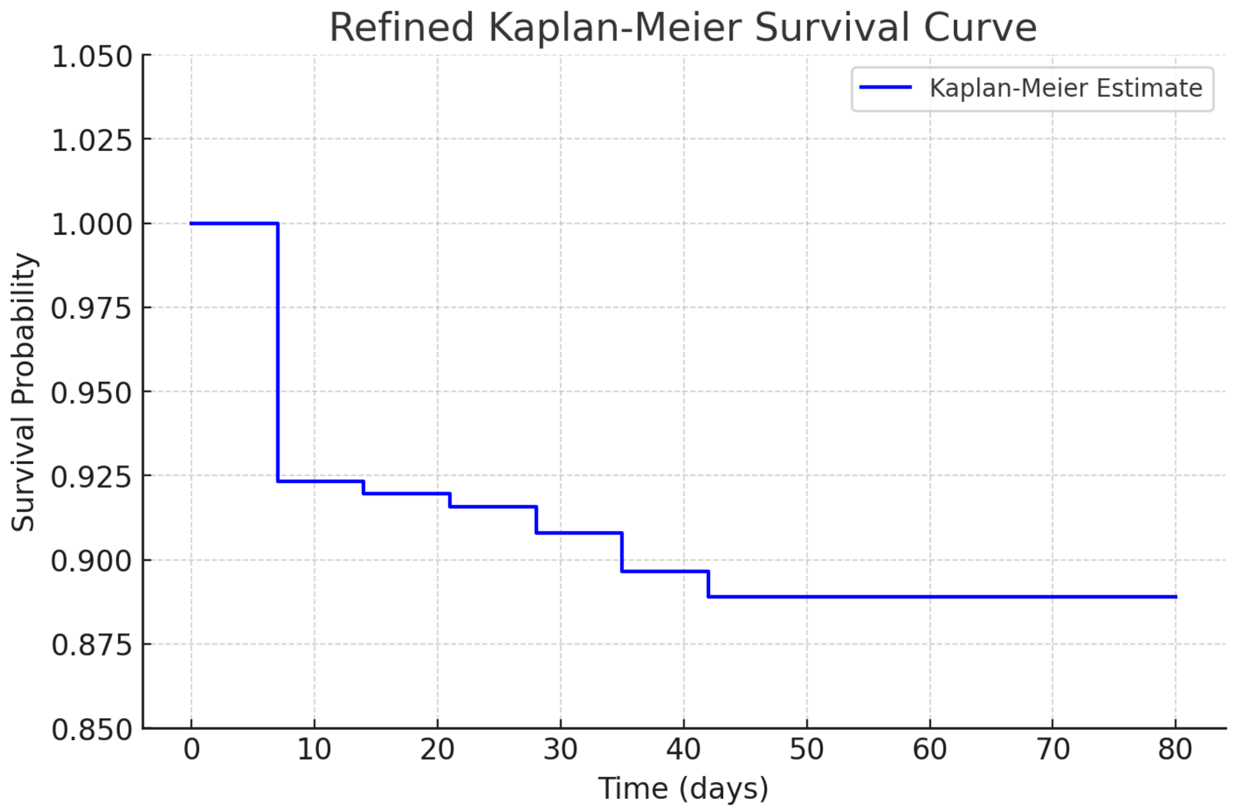

Figure 1. Kaplan-Meier survival curve for participants with acute non-traumatic abdominal pain, illustrating the time-to-death distribution. The survival probability declined gradually, with most events occurring within the first 7days of admission, and then progressively stabilizing around 88%.
